# Protocol for immunostaining of non-adherent cells and cellular structures using centrifugal filter devices

**DOI:** 10.1016/j.xpro.2025.103934

**Published:** 2025-07-04

**Authors:** Momoko Miyazawa, Kentaro Kawai, Shohei Yamamoto, Daiju Kitagawa

**Affiliations:** 1 Department of Physiological Chemistry, Graduate School of Pharmaceutical Sciences, The University of Tokyo, Bunkyo, Tokyo 113-0033, Japan

**Keywords:** Cell Biology, Molecular Biology, Protein Biochemistry

## Abstract

Immunostaining of non-adherent cells can be technically challenging due to the difficulty of efficiently exchanging solutions, a crucial process during immunostaining. Here, we present a protocol that utilizes centrifugal filter devices for immunostaining of non-adherent cells. We describe instructions for simple and efficient solution exchanges during the staining procedure. We also illustrate an application for staining isolated organelles, such as centrosomes. Overall, this protocol provides a practical technique for immunostaining a wide variety of cell types and cellular structures.

## Highlights

• Guidance on centrifugal filter-based immunostaining
• Instructions for immunostaining of non-adherent cells and cellular structures
• Applicable for staining various cell types and organelles

## Abstract (editor highlights)

Publisher’s note: Undertaking any experimental protocol requires adherence to local institutional guidelines for laboratory safety and ethics.

## Graphical abstract

**Figure:**
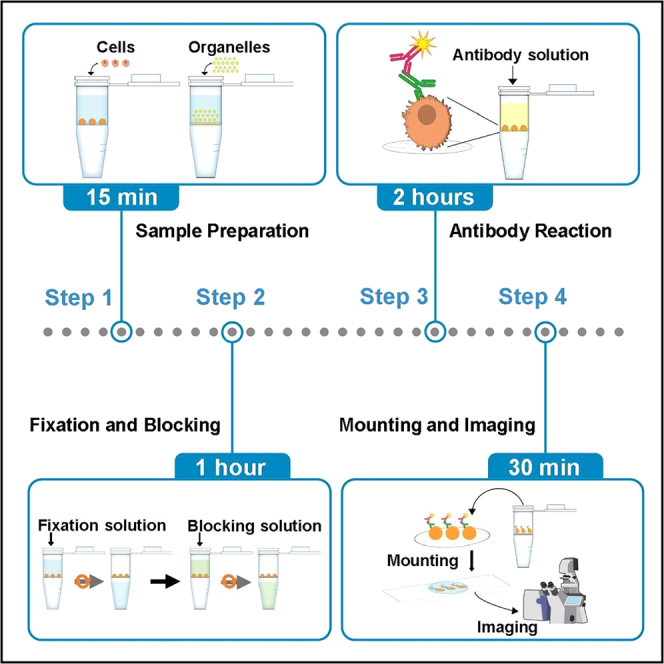

## Before you begin

Immunostaining of adherent cells is typically facilitated by their ability to attach to substrates such as glass. This adhesion allows for easy exchange of solutions, including fixation solutions and antibodies. However, immunostaining becomes challenging for certain cell types due to their lack of adhesive properties. To date, immunostaining of non-adherent cells has commonly been achieved through immobilizing the cells by centrifuging them onto substrates using specialized equipment like Cytospin, or artificially inducing adhesion through coatings of substrates with chemical compounds.^1^^,^^2^^,^^3^^,^^4^ Similar challenges arise when attempting to immunostain cellular structures in cell lysate or isolated from cells.^5^^,^^6^^,^^7^ Here, we present an alternative approach for immunostaining non-adherent cells using centrifugal filter devices equipped with a polyvinylidene fluoride (PVDF) membrane. The devices capture cells or cellular structures on the filter membranes while allowing the exchange of solutions necessary for immunostaining. This method eliminates the need for specialized machines like Cytospin or additional reagents, making it more accessible.

In this protocol, we demonstrate the immunostaining of K562 and Jurkat cells, human leukemia cell lines, and HUDEP-2, a human erythroid progenitor cell line, as representative models of non-adherent cells.^8^^,^^9^^,^^10^ Additionally, we present the staining of suspended HeLa cells, an adherent cell line, indicating its potential application for analyzing adherent cell suspensions, for example, during cell sorting experiments or when cell adhesion is affected. As an example of cellular structures, we also show the staining of isolated centrosomes from mitotic HeLa and RPE-1 cells.

### Preparation of K562, Jurkat, and HUDEP-2 cells

1. Maintain K562 cells in IMDM supplemented with 10% FBS and 1% penicillin/streptomycin. For Jurkat cells, use RPMI-1640 supplemented with 10% FBS and 1% penicillin/streptomycin. For HUDEP-2 cells, use StemSpan SFEM supplemented with 1 μg/mL Dox, 1 μM of Dex, 50 ng/mL of SCF, 3 U/mL EPO and 1% penicillin/streptomycin. Culture cells at 37°C in 5% CO_2_ atmosphere.
2. Collect cells from cell culture.
3. Determine cell concentrations using a hemocytometer.

### Preparation of mitotic HeLa and RPE-1 cells

4. Maintain HeLa cells in DMEM-High glucose supplemented with 10% FBS and 1% penicillin/streptomycin. For RPE-1 cells, use DMEM/Ham’s F-12 supplemented with 10% FBS and 1% penicillin/streptomycin. Culture cells at 37°C in 5% CO_2_ atmosphere. For passaging, detach cells from culture dishes using trypsin-EDTA solution.
5. Cell cycle synchronization.
  a. Add Nocodazole to the culture medium at a final concentration of 150 nM for HeLa cells and 300 nM for RPE-1 cells.
  b. Incubate at 37°C in 5% CO_2_ atmosphere for 12–15 h.
  c. Detach mitotic cells from the culture dishes by shaking the dishes.
6. Collect the culture medium containing detached mitotic cells.
  a. Centrifuge at 200 g for 2 min.
  b. Remove the supernatant.
  c. Resuspend the cells with D-PBS (1.25 mL for cells from 1× 90 mm dish).
  d. Centrifuge at 200 g for 2 min.
  e. Remove the supernatant.
  f. Resuspend the cells in the culture medium (1.25 mL for cells from 1× 90 mm dish).
7. Incubate at 37°C in 5% CO_2_ atmosphere for 30 min to release cells from nocodazole-induced arrest.
8. Determine cell concentrations using a hemocytometer.

## Key resources table

**Table:** 

REAGENT or RESOURCE	SOURCE	IDENTIFIER
**Antibodies**		

Mouse anti-CD71 (1:400 dilution)	Proteintech	Cat# 66180-1-Ig, RRID: AB_2881575
Mouse anti-Centrin clone 20H5 (1:500 dilution)	Millipore	Cat# 04-1624, RRID: AB_10563501
Mouse anti-IAK1(Aurora A) (1:250 dilution)	BD Biosciences	Cat# 610938, RRID: AB_398251
Mouse anti-α-tubulin (3:1,000 dilution)	Sigma-Aldrich	Cat#T5168, RRID: AB_477579
Mouse anti-β-actin (1:100 dilution)	Santa Cruz Biotechnology	Cat# sc-47778, RRID: AB_626632
Rabbit anti-CDK5RAP2 (1:500 dilution)	Bethyl Laboratories	Cat# IHC-00063, RRID: AB_2076863
Rabbit anti-Pericentrin (1:2,000 dilution)	Abcam	Cat#ab4448, RRID: AB_304461
Rabbit anti-TGN46 (1:1,000 dilution)	Proteintech	Cat# 13573-1-AP, RRID: AB_10597396
Rabbit anti-α-Tubulin (3:1,000 dilution)	MBL	Cat# PM054, RRID: AB_10598496
Rat anti-Centrin 2 (1:500 dilution)	BioLegend	Cat#698601, RRID: AB_2715793
Rabbit anti-γ-Tubulin (1:1,000 dilution)	Sigma-Aldrich	Cat# T5192, RRID: AB_261690
Guinea pig anti-α-Tubulin (1:1,000 dilution)	ABCD Antibodies	Cat# ABCD_AA345, RRID: AB_3106979
Donkey anti-mouse IgG (H+L) secondary antibody, Alexa Fluor Plus 555 (1:1,000–1:2,000 dilution)	Thermo Fisher Scientific	Cat# A32773, RRID: AB_2762848
Donkey anti-mouse IgG (H+L) secondary antibody, Alexa Fluor Plus 647 (1:1,000 dilution)	Thermo Fisher Scientific	Cat# A32787, RRID: AB_2762830
Donkey anti-rabbit IgG (H+L) secondary antibody, Alexa Fluor Plus 488 (1:1,000 dilution)	Thermo Fisher Scientific	Cat# A32790, RRID: AB_2762833
Donkey anti-rabbit IgG (H+L) secondary antibody, Alexa Fluor Plus 555 (1:1,000 dilution)	Thermo Fisher Scientific	Cat# A32794, RRID: AB_2762834
Donkey anti-rabbit IgG (H+L) secondary antibody, Alexa Fluor Plus 647 (1:1,000–1:2,000 dilution)	Thermo Fisher Scientific	Cat# A32795, RRID: AB_2762835
Donkey anti-rat IgG (H+L) secondary antibody, Alexa Fluor Plus 555 (1:2,000 dilution)	Thermo Fisher Scientific	Cat# A48270, RRID: AB_2896336
Goat anti-guinea pig IgG (H+L) secondary antibody, Alexa Fluor 488 (1:1,000 dilution)	Molecular Probes	Cat# A-11073, RRID: AB_2534117

**Chemicals, peptides, and recombinant proteins**		

Sodium chloride	Nacalai	31319-45
Potassium chloride	Wako	163-03545
Di-sodium hydrogen phosphate 12-water	Nacalai	31722-45
Potassium dihydrogen phosphate	Wako	164-22635
Dulbecco’s modified Eagle’s medium (DMEM)/high glucose	Nacalai	08459-64
DMEM/Ham’s F-12	Nacalai	11581-15
Iscove’s modified Dulbecco’s medium (IMDM) with L-glutamine and HEPES	Nacalai	11506-05
Roswell Park Memorial Institute 1640 medium (RPMI-1640)	Nacalai	30264-85
StemSpan SFEM	STEMCELL Technologies	ST-09650
Fetal bovine serum (FBS)	Gibco	10270-106
Dulbecco’s phosphate-buffered saline (D-PBS)	Nacalai	14249-24
Penicillin-streptomycin mixed solution	Nacalai	09367-34
Trypsin-EDTA solution	Nacalai	32778-05
Doxycycline (Dox)	Tokyo Chemical Industry	D4116
Dexamethasone (Dex)	Nacalai	11107-51
Stem cell factor (SCF)	Nacalai	21065-04
Erythropoietin (EPO)	Chugai Pharmaceutical Co., Ltd.	4987136117986
Methanol	Nacalai	21914-03
4% paraformaldehyde (PFA)/phosphate buffer solution	Nacalai	09154-56
Triton X-100	Nacalai	35501-15
Glycerol	Wako	075-00611
Bovine serum albumin (BSA)	Sigma-Aldrich	A9647-100G
Hoechst 33258	Nacalai	19173-41
Nocodazole	Sigma-Aldrich	M1404-10MG
Cytochalasin D	Wako	034-25881
Dimethyl sulfoxide	Nacalai	09659-14
2-mercaptoethanol (2-ME)	Nacalai	21418-42
Protease inhibitor cocktail	Nacalai	25955-11
Tris(hydroxymethyl)aminomethane (Tris)	Nacalai	35434-21
Hydrochloric acid (HCl)	Nacalai	18320-15
Calcium chloride (CaCl_2_)	Sigma	C1016
Magnesium chloride hexahydrate (MgCl_2_・6H_2_O)	Nacalai	20909-55
HEPES	Nacalai	02443-05
PIPES	Nacalai	28104-16
Nonidet P40 substitute (NP-40)	Nacalai	25223-75
DNase I	Takara	2270A
Sucrose, applicable for density-gradient centrifugation	Nacalai	30406-25
Sodium azide	Wako	195-11092
Potassium hydroxide (KOH)	Wako	168-21815

**Experimental models: Organisms/strains**		

K562	RIKEN BRC	RCB0027
Jurkat	ATCC	TIB-152
HUDEP-2	RIKEN BRC	RCB4557
HeLa	ECACC	93021013
RPE-1	ATCC	CRL-4000

**Other**		

Ultrafree-MC centrifugal filter, 0.1 μm pore size, PVDF	Millipore	UFC30VV00
Ultrafree-MC centrifugal filter, 0.22 μm pore size, PVDF	Millipore	UFC30GV00
Ultrafree-MC centrifugal filter, 0.45 μm pore size, PVDF	Millipore	UFC30HV00
Ultrafree-MC centrifugal filter, 0.65 μm pore size, PVDF	Millipore	UFC30DV0S
Cover glass, 12 mm (thickness: 0.13–0.17 mm)	Matsunami	C012001
Cover glass, 15 mm (thickness: 0.13–0.17 mm)	Matsunami	C015001
Glass slide	Matsunami	S0318
60 mm cell culture dishes	SPL Life Sciences	20060
90 mm cell culture dishes	NIPPON Genetics	FG-2090
1.5 mL tubes	Watson	131-815C
Table top centrifuge machine	Kubota	3520
Table top centrifuge machine	Eppendorf	5702
Beckman open-top thick-wall polycarbonate tubes	Beckman	347287
Beckman Optima TLX	Beckman	N/A
Beckman TLA-100.4 fixed angle rotor	Beckman	N/A
Beckman TLA-120.2 fixed angle rotor	Beckman	357656
Tweezers	KFI	1-9749-22
Scissors	PLUS Corporation	SC-165S
Nail polish	N/A	N/A
Plastic bags	N/A	N/A
Hemocytometer	N/A	N/A
Micropipette	N/A	N/A
Confocal microscope equipped with a 63× oil-immersion objective (NA 1.4)	Leica TCS SP8	N/A
Fluorescence microscope equipped with a 63× oil-immersion objective (NA 1.4)	Carl Zeiss, Axioplan 2	N/A

## Materials and equipment

**Table:** 10× Phosphate-buffered saline (10× PBS)

Reagent	Final concentration	Amount
Sodium chloride	1.37 M	80 g
Potassium chloride	27 mM	2 g
Di-sodium hydrogen phosphate 12-water	100 mM	35.8 g
Potassium dihydrogen phosphate	18 mM	2.4 g
dH_2_O	N/A	1 L

Note on storage conditions: The solution can be stored at 20°C–25°C for months.

**Table:** 1× Phosphate-buffered saline (1× PBS)

Reagent	Final concentration	Amount
10× Phosphate-buffered saline	1×	1 L
dH_2_O	N/A	9 L

Note on storage conditions: The solution can be stored at 20°C–25°C for months.

**Table:** 10 mM Nocodazole

Reagent	Final concentration	Amount
Nocodazole	10 mM	10 mg
Dimethyl Sulfoxide	N/A	3.32 mL

Note on storage conditions: The solution can be stored at −20°C for months.

**Table:** Blocking solution

Reagent	Final concentration	Amount
BSA	1% (w/v)	10 g
Triton X-100	0.05% (v/v)	500 μL
Sodium azide	0.03% (w/v)	0.3 g
1× PBS	N/A	1 L

Note on storage conditions: The solution can be stored at 4°C for months.

**Table:** Mounting solution

Reagent	Final concentration	Amount
Glycerol	90%	90 mL
1× PBS	N/A	10 mL

Note on storage conditions: The solution can be stored at 20°C–25°C for months.

**Table:** 2 mM Cytochalasin D

Reagent	Final concentration	Amount
Cytochalasin D	2 mM	1 mg
Dimethyl Sulfoxide	N/A	985 mL

Note on storage conditions: The solution can be stored at −20°C for months.

**Table:** 10× Washing buffer for centrosome isolation

Reagent	Final concentration	Amount
Tris	20 mM	24.2 mg
CaCl_2_	2 mM	2.22 mg
KCl	500 mM	373 mg
MgCl_2_・6H_2_O	20 mM	40.7 mg
10 N HCl	N/A	add dropwise until pH 8.0
dH_2_O	N/A	Up to 10 mL

Note on storage conditions: The solution can be stored at 4°C for months.

**Table:** 1× Washing buffer for centrosome isolation

Reagent	Final concentration	Amount
10× Washing buffer for centrosome isolation	1×	100 μL
2-Mercaptoethanol	0.1% (v/v)	1 μL
Protease inhibitor cocktail	1:1000	1 μL
2 mM Cytochalasin D	2 μM	1 μL
dH_2_O	N/A	897 μL

Note on storage conditions: The solution should be prepared just before use.

**Table:** 1 M K-HEPES (pH 7.5)

Reagent	Final concentration	Amount
HEPES	1 M	11.9 g
10N KOH	N/A	add dropwise until pH 7.5
dH_2_O	N/A	Up to 50 mL

Note on storage conditions: The solution can be stored at −20°C for months.

**Table:** Lysis buffer for centrosome isolation

Reagent	Final concentration	Amount
1 M K-HEPES (pH 7.5)	1 mM	1 μL
NP-40	0.5% (v/v)	5 μL
1 M MgCl_2_	0.5 mM	0.5 μL
2-Mercaptoethanol	0.1% (v/v)	1 μL
Protease inhibitor cocktail	1:1000	1 μL
dH_2_O	N/A	991.5 μL

Note on storage conditions: The solution should be prepared just before use.

**Table:** 10 mM K-PIPES (pH 7.2)

Reagent	Final concentration	Amount
PIPES	10 mM	0.30 g
10N KOH	N/A	add dropwise until pH 7.2
dH_2_O	N/A	Up to 100 mL

Note on storage conditions: The solution can be stored at −20°C for months.

**Table:** 70% (w/w) sucrose solution

Reagent	Final concentration	Amount
Sucrose	70% (w/w)	28 g
10 mM K-PIPES (pH 7.2)	N/A	12 g

Note on storage conditions: The solution should be prepared just before use.

***Note:*** To other concentrations of sucrose solution (35, 50, 60%), dilute 70% (w/w) sucrose solutions with the 10 mM K-PIPES (pH 7.2). Before use, add 0.1% 2-Mercaptoethanol and Protease inhibitor cocktail (at 1:1000 dilution).

## Step-by-step method details

### Immunostaining of non-adherent cells using centrifugal filter devices

**Timing: 3 h**

As examples of non-adherent or suspended adherent cells, we present the immunostaining procedure using K562, Jurkat, HUDEP-2 and HeLa cells.

**CRITICAL:** Avoid drying out the filter membrane during immunostaining as it can affect the specific binding of antibodies and increase background noise.

**Figure 1:**
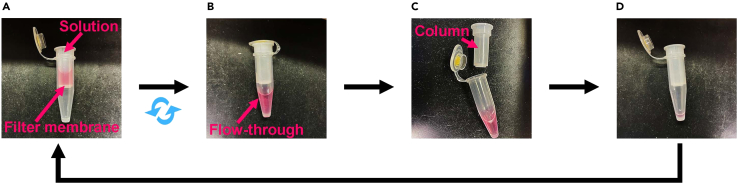
Solution exchange using centrifugal filter devices The solution exchange process for immunostaining. Cells or cellular structures larger than the pore size of the filter are retained on the filter membrane after centrifugation, enabling multiple washes, fixation, blocking, and antibody incubation. The steps for solution exchange are as follows: (A and B) Add solution into the column and centrifuge it. (C and D) Remove the flow-through after centrifugation.

1. Collection of cells on the filter.
  a. Apply 100 μL of the cell suspension (5.0 × 10^4^ cells for K562, Jurkat and HUDEP-2 cells, 2.0 × 10^4^ cells for HeLa cells) onto an Ultrafree-MC column with a 0.65 μm pore size PVDF membrane (Figure 1).
    ***Note:*** Adjust cell number for optimal observation and analysis. Selecting a cell number on the order of 10^4^ would be better for efficient observation and to avoid filter clogging.
    ***Optional:*** 0.45 μm pore size PVDF membrane can be used as well. In this case, increase the centrifugation speed to 300 g to allow the buffers to flow through.
    ***Optional:*** Alternatively, Ultrafree-CL columns, which offer a larger filter membrane area, can be used to enhance the throughput.
    ***Optional:*** Washing cells with 1× PBS prior to loading onto the columns might improve the quality of staining by removing cell debris in the culture medium.
  b. Add 400 μL of 1× PBS.
  c. Centrifuge at 100 g for 1 min and discard the flow-through (troubleshooting problem 1).
2. Fixation of cells.
  a. PFA fixation: Add 300 μL of 4% PFA solution and incubate at 20°C−25°C for 30 min.
    Methanol fixation: Add 300 μL of cold methanol (−20°C) and incubate at −20°C for 5 min.
    ***Note:*** Choose optimal fixation conditions depending on the specific targets and the antibodies used.
  b. Centrifuge at 100 g for 1 min and discard the flow-through.
  c. Add 300 μL of 1× PBS.
  d. Centrifuge at 100 g for 1 min and discard the flow-through.
  e. Add 300 μL of 1× PBS.
  f. Centrifuge at 100 g for 1 min and discard the flow-through.
    **Pause point:** It is possible to keep the samples in PBS at 4°C for at least 1 day after fixation and washing (troubleshootingproblem 2).
3. Blocking.
  a. Add 200 μL of blocking solution and incubate at 20°C−25°C for 30 min (troubleshooting problem 2).
    ***Optional:*** Consider the composition of blocking solution and the incubation time to minimize non-specific binding of the antibodies used.
  b. Centrifuge at 100 g for 1 min and discard the flow-through.
4. Incubation with primary antibodies.
  a. Add 100 μL of primary antibody solution diluted in blocking solution and incubate at 20°C−25°C for 60 min (troubleshooting problem 2).
    ***Note:*** Optimize antibody concentrations and incubation time based on antibody specificity and experimental outcome.
  b. Centrifuge at 100 g for 1 min and discard the flow-through.
  c. Add 300 μL of 1× PBS.
  d. Centrifuge at 100 g for 1 min and discard the flow-through.
  e. Add 300 μL of 1× PBS.
  f. Centrifuge at 100 g for 1 min and discard the flow-through.
5. Incubation with secondary antibodies.
  a. Add 100 μL of secondary antibody solution diluted in the blocking solution and incubate at 20°C−25°C for 30 min (troubleshooting problem 2).
    **CRITICAL:** To prevent fluorescence fading during sample preparation, prepare and incubate the sample with the secondary antibodies in the dark.
    ***Note:*** Optimize antibody concentrations and incubation time based on antibody specificity and experimental outcome.
  b. Centrifuge at 100 g for 1 min and discard the flow-through.
  c. Add 300 μL of 1× PBS.
  d. Centrifuge at 100 g for 1 min and discard the flow-through.
  e. Add 300 μL of 1× PBS.
  f. Centrifuge at 100 g for 1 min and discard the flow-through.
    **CRITICAL:** Immediately after the final wash, proceed to mounting as described in the following section (step 12-a).

### Immunostaining of isolated centrosomes using centrifugal filter devices

**Timing: 7 h**

Here, we present the immunostaining procedure for centrosomes isolated from mitotic HeLa and RPE-1 cells. The cellular structures larger than 0.1 μm, such as centrosomes, would be effectively retained on the filter membrane, allowing solution exchanges during immunostaining. The centrosomes were collected as previously described.^11^

**CRITICAL:** Avoid drying out the filter membrane during immunostaining as it can affect the specific binding of antibodies and increase background noise.

6. Isolation of centrosomes.
  a. Cell lysis.
    i. Following the preparation of mitotic HeLa or RPE-1 cells (1 × 10^7^ cells) as described in the “before you begin” section, incubate the cells with 2 μM Cytochalasin D at 37°C for 20 min.
    ii. Centrifuge the cells at 500 g for 2 min.
    iii. Resuspend the cell pellet in 4 mL of 1× PBS containing 2 μM Cytochalasin D and centrifuge at 500 g for 2 min. Repeat this wash step once.
    iv. Resuspend the cell pellet in 1 mL of washing buffer.
      **CRITICAL:** Keep samples on ice or at 4°C from this step.
    v. Centrifuge the cells at 500 g for 2 min at 4°C and carefully remove the supernatant.
    vi. Resuspend the cell pellet in 1 mL of lysis buffer and rotate the tube at 4°C for 45 min.
    vii. Centrifuge at 5,000 g for 2 min at 4°C and carefully collect the supernatant.
    viii. Add DNase I to a final concentration of 2 units/mL and incubate the tube at 4°C for 30 min with gentle rotation.
  b. Isolation.
    i. Carefully layer 160 μL of 60% sucrose solution at the bottom of an ultracentrifuge tube. Gently overlay the cell lysate (approximately 950 μL) onto the sucrose cushion.
    ii. Centrifuge at 25,000 g at 4°C for 30 min using an ultracentrifuge.
    iii. Carefully discard the top 630 μL of the supernatant.
    iv. Gently mix the remaining lysate with the sucrose cushion in the tube.
    v. Prepare a sucrose density gradient in a new ultracentrifuge tube by slowly layering 400 μL of each sucrose solution in the following order (from bottom to top): 70%, 50% and 35%.
    vi. Gently load the mixture from step (6-b-iv) onto the top of the prepared sucrose gradient.
    vii. Centrifuge at 130,000 g at 4°C for 90 min using an ultracentrifuge.
    viii. Carefully fractionate and collect samples from the top 150 μL/fraction using a micropipette.
      ***Note:*** The centrosomes tend to be enriched in the fraction 4 to 6 from the bottom. Confirm the centrosome-enriched fractions by immunostaining as described below or western blotting.^11^
      **Pause point:** Samples can be cryopreserved at −80°C following flash-freezing in liquid nitrogen for at least 2 months.
7. Capture of isolated centrosomes on the filter.
  a. Mix 15–30 μL of the fraction of interest containing centrosomes with 400 μL of 1× PBS.
  b. Transfer the centrosome suspension to an Ultrafree-MC column with a 0.1 or 0.22 μm pore size PVDF membrane (Figure 1).
    ***Note:*** Choose an appropriate pore size based on the sizes of the target cellular structures and experimental needs.
  c. Centrifuge at 3000 g for 1 min and discard the flow-through (troubleshooting problem 1).
8. Fixation.
  a. Add 350 μL of the cold methanol (−20°C).
    ***Note:*** Choose optimal fixation conditions depending on the specific targets and the antibodies used.
  b. Incubate on ice for 3 min.
  c. Centrifuge at 3000 g for 1 min and discard the flow-through.
  d. Add 400 μL of 1× PBS.
  e. Centrifuge at 3000 g for 1 min and discard the flow-through.
  f. Add 400 μL of 1× PBS.
  g. Centrifuge at 3000 g for 1 min and discard the flow-through.
    **Pause point:** It is possible to keep the samples in PBS at 4°C for at least 1 day after fixation and washing (troubleshootingproblem 2).
9. Blocking.
  a. Add 400 μL of blocking solution and incubate at 20°C−25°C for 15 min.
    ***Optional:*** Consider the composition of blocking solution and the incubation time to minimize non-specific binding of the antibodies used.
  b. Centrifuge at 3000 g for 1 min and discard the flow-through.
10. Incubation with primary antibodies.
  a. Add 100 μL of primary antibody solution diluted in blocking solution and incubate at 20°C−25°C for 60 min (troubleshooting problem 2).
    ***Note:*** Optimize antibody concentrations and incubation time based on antibody specificity and experimental outcome.
  b. Centrifuge at 3000 g for 1 min and discard the flow-through.
  c. Add 400 μL of 1× PBS.
  d. Centrifuge at 3000 g for 1 min and discard the flow-through.
  e. Add 400 μL of 1× PBS.
  f. Centrifuge at 3000 g for 1 min and discard the flow-through.
11. Incubation with secondary antibodies.
  a. Add 100 μL of secondary antibody solution and incubate at 20°C−25°C for 30 min (troubleshooting problem 2).
    **CRITICAL:** To prevent fluorescence fading during sample preparation, prepare and incubate the sample with the secondary antibodies in the dark.
    ***Note:*** Optimize antibody concentrations and incubation time based on antibody specificity and experimental outcome.
  b. Add 400 μL of 1× PBS.
  c. Centrifuge at 3000 g for 1 min and discard the flow-through.
  d. Add 300 μL of 1× PBS.
  e. Centrifuge at 3000 g for 1 min and discard the flow-through.
    Immediately after the final wash, proceed to mounting as described in the following section (step 12-a).

### Mounting of the stained samples

**Timing: 15–30 min**

**Figure 2:**
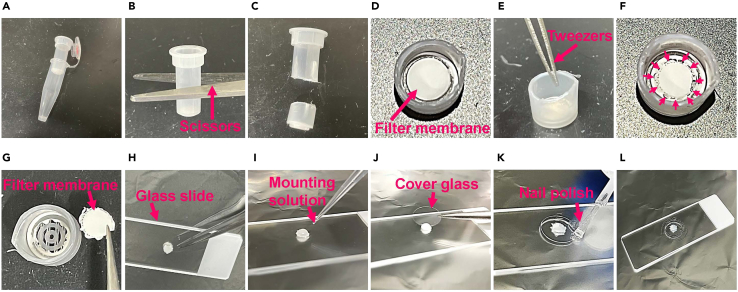
Mounting procedure Step-by-step images of the mounting procedure, with numbers corresponding to the steps in the ‘mounting of the stained samples’ section (step 12 and 13). (A) Remove the column from the centrifugal filter device *(12-a)*. (B and C) Cut the column with scissors *(12-b)*. (D) Top view of the bottom-side of the column. (E and F) Poke holes around the edges of the filter membrane with tweezers. Red arrows indicate the holes *(12-c)*. (G) Lift the filter membrane *(12-d)*. (H) Place the filter membrane onto a glass slide with the sample side facing up *(12-d)*. (I) Put the mounting solution onto the filter membrane *(13-a)*. (J) Place a cover glass onto the filter membrane *(13-b)*. (K) Seal the edges of the cover glass with nail polish *(13-c)*. (L) Completed specimen.

Following the final wash, the filter membranes are carefully removed from the columns and mounted for imaging (Figure 2 and Methods video S1).

**CRITICAL:** Avoid drying out the filter membrane during the mounting process as it can affect the specific binding of antibodies and increase background noise.

12. Collection of the filter membrane from the column.
  a. Immediately after the last centrifugation, remove the column from the centrifugal filter device.
  b. Cut the column in the middle using scissors.
    ***Note:*** Cut the column inside a plastic bag to prevent the column piece from flying away.
    ***Note:*** Ordinary scissors are sufficient to cut the column, but the sharpness of the blades will affect the cutting efficiency.
  c. Carefully cut around the edges of the filter membrane with the tip of tweezers.
  d. Carefully lift the filter membrane using tweezers and place it onto a glass slide with the sample side facing up.
    ***Note:*** Carefully grasp the edge of the filter using tweezers to avoid damaging cells on the membrane.
13. Mounting.
  a. Put 10–20 μL of mounting solution onto the filter membrane.
    ***Note:*** For 12 mm and 15 mm cover glasses, use 10–15 μL and 15–20 μL of mounting solution, respectively.
  b. Place the cover glass gently over the filter membrane.
  c. Seal the edges of the cover glass with nail polish to immobilize it.
  d. Observe the sample using a fluorescence microscope (troubleshooting problem 3, 4, 5, 6 and 7).
    ***Note:*** Mounted samples can be stored at 4°C in the dark for at least 1 week.

Methods video S1. Mounting procedure, related to steps 12 and 13 The procedure corresponds to the steps described in the 'Mounting of the stained samples' section (step 12 and 13).

## Expected outcomes

**Figure 3:**
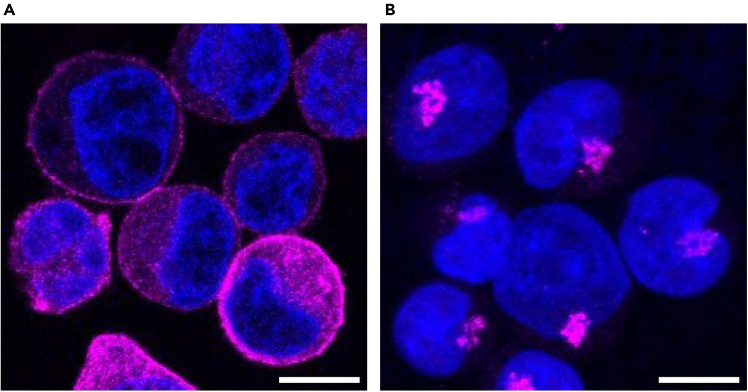
Confocal microscopic images of K562 cells (A and B) K562 cells were fixed with 4% PFA and stained with antibodies against CD71 (Magenta), a cell surface marker, and TGN46 (Magenta), a Golgi marker, respectively. DNA (Blue) was stained with Hoechst 33258. The images were acquired using a confocal microscope. A single slice image (A) and a maximum intensity projection image (B) are shown. Scale bar: 10 μm.

**Figure 4:**
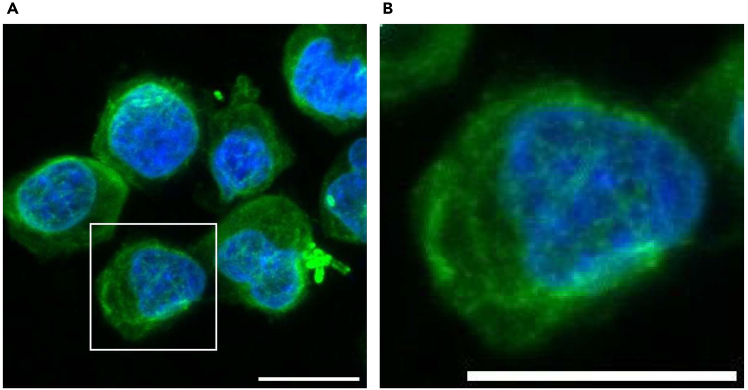
Confocal microscopic images of Jurkat cells (A) Jurkat cells were fixed with 4% PFA and stained with antibodies against α-tubulin (Green) and Hoechst 33258 (Blue). (B) A magnified view of the indicated region (white box) in (A) is shown. The images were acquired using a confocal microscope. The maximum intensity projection images are shown. Scale bar: 10 μm.

**Figure 5:**
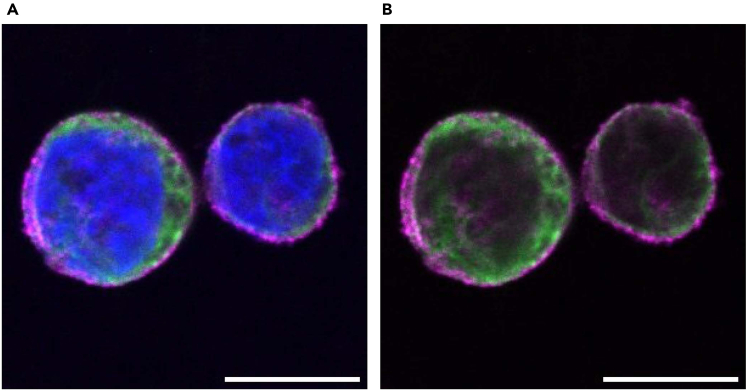
Confocal microscopic images of HUDEP-2 cells (A and B) HUDEP-2 cells were fixed with cold methanol and stained with antibodies against α-tubulin (Green), β-actin (Magenta) and Hoechst 33258 (Blue). (B) shows the same field of view as (A), but without the DNA signal (blue) to highlight the distribution of actin and tubulin. The images were acquired using a confocal microscope. Single slice images are shown. Scale bar: 10 μm.

**Figure 6:**
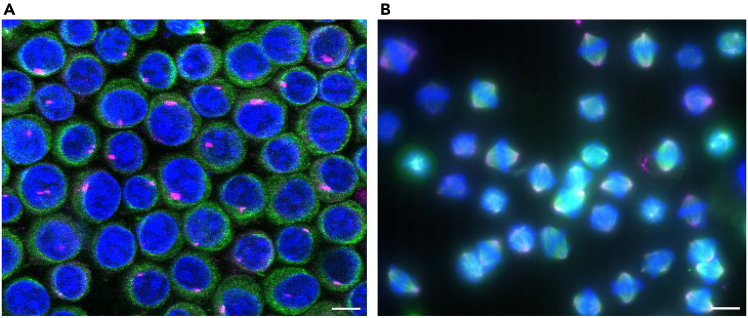
Immunostaining of suspended HeLa cells (A) Asynchronously growing HeLa cells, which were cultured without synchronization and detached from culture dishes by trypsinization, were fixed with cold methanol. The cells were stained with antibodies against Pericentrin (Magenta), a centrosome marker and α-tubulin (Green). (B) Mitotic HeLa cells were collected by shake-off and fixed with cold methanol. The cells were stained with antibodies against Aurora A (Magenta) and α-tubulin (Green). DNA (Blue) was visualized with Hoechst 33258. The images were acquired using a confocal microscope (A) or a conventional fluorescence microscope (B). Maximum intensity projected images are shown. Scale bar: 10 μm.

This protocol is applicable to a variety of cell types and cellular structures. Here, we present some examples of immunostained samples visualized using a confocal microscope or a conventional fluorescence microscope. The data demonstrate that blood cell types including K562, Jurkat and HUDEP-2 cells can be immunostained using this protocol (Figures 3, 4, 5, and 6). As previously reported, CD71, a cell surface protein, is detected on the cell surface of the K562 cells (Figure 3A).^12^ In addition, TGN46, a Golgi marker protein, is also detected in K562 cells, showing membrane compartments inside cells (Figure 3B).^13^ Cytoskeleton networks in Jurkat and HUDEP-2 cells are also clearly visualized (Figures 4 and 5).

This protocol is expected to be applied for immunostaining of suspended adherent cells, for example: when cell adhesion is chemically or genetically affected; when analyzing cell suspensions during cell sorting; or when analyzing weakly adherent cells that are difficult to immunostain while attached. As examples of staining adherent cell suspensions, we present immunostaining of HeLa cells. Asynchronously growing HeLa cells, detached by trypsinization, exhibit round cell shapes and interphase nuclei (Figure 6A). Mitotic HeLa cells, collected after cell cycle synchronization and shake-off, display mitotic spindles and chromosomes (Figure 6B).^14^

**Figure 7:**
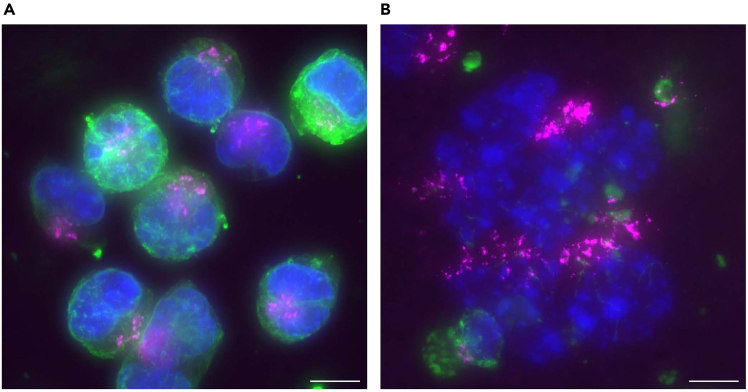
K562 cells disrupted by high-speed centrifugation during immunostaining K562 cells were fixed with 4% PFA and stained with antibodies against α-tubulin (Green), TGN46 (Magenta), a Golgi marker, and Hoechst 33258 (Blue). Immunostaining was performed with centrifugation speed at 100 g (A) or 3,000 g (B). As shown in (B), the higher centrifugation speed (3,000 g) during immunostaining disrupted cell and nuclear morphologies, showing dispersed nuclei and intracellular structures. The image was acquired using a conventional fluorescence microscope. Maximum intensity projected images are shown. Scale bar: 10 μm.

**Figure 8:**
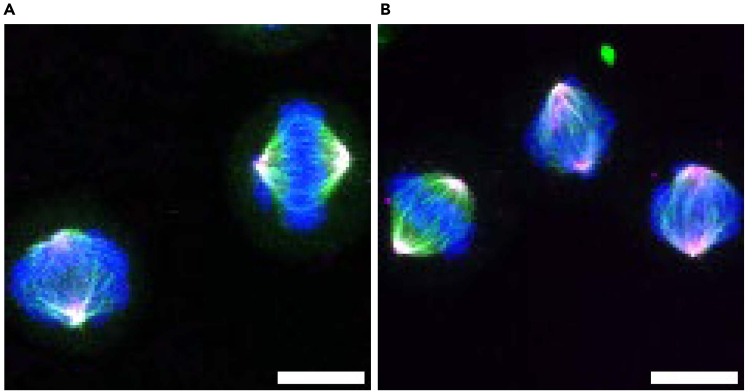
Mitotic spindle structures in HeLa cells Mitotic HeLa cells were immunostained using this protocol (A) or a conventional method (B). The cells were fixed with cold methanol and stained with antibodies against Aurora A (Magenta), α-tubulin (Green) and Hoechst 33258 (Blue). (A) Cells were collected by mitotic shake-off and immunostained with a centrifugal filter column. Centrifugation during immunostaining was conducted at 300 g. (B) Cells were seeded onto cover glasses and the adhered cells were conventionally immunostained without cell detachment. The images were acquired using a confocal microscope. Maximum intensity projected images are shown. Scale bar: 10 μm.

During immunostaining, performing centrifugation at lower speeds (up to 300 g) is recommended to maintain the normal morphology of cells and their nuclei (Figure 7A). Higher speed centrifugation can significantly affect cell morphologies as evidenced by the presence of disrupted nuclei and dispersed Golgi (TGN-46) and microtubule (α-tubulin) signals in K562 cells (Figure 7B). The lower centrifugation speed appears to preserve the structures of mitotic spindles in HeLa cells (Figure 8A), showing comparability with a conventional immunostaining method (Figure 8B).^14^

**Figure 9:**
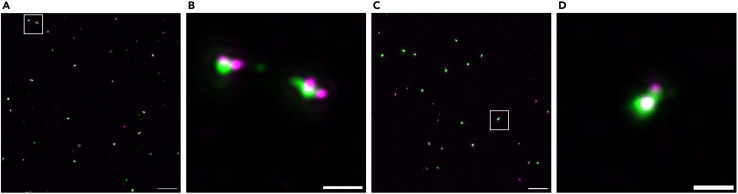
Confocal microscopic images of mitotic centrosomes isolated from HeLa and RPE-1 cells (A and B) Mitotic centrosomes isolated from HeLa cells were fixed with cold methanol and stained with the indicated antibodies. CDK5RAP2 (Green) and Centrin (Magenta) were visualized as centrosome markers. (C and D) Mitotic centrosomes isolated from RPE-1 cells were fixed with cold methanol and stained with antibodies against γ-tubulin (Green) and Centrin (Magenta). Magnified views of the indicated regions (white boxes) in (A and C) are shown in (B and D), respectively. The images were acquired using a confocal microscope. Deconvolution of images was performed using Huygens essential software. The maximum intensity projection images are shown. Scale bars: 5 μm (A and C), 1 μm (B and D).

As an example of isolated cellular structures, we present the staining of mitotic centrosomes isolated from HeLa and RPE-1 cells. The preservation of centrosomes is suggested by the co-localization of centrosome protein signals, which include two pairs of centrioles (indicated by centrin signals) surrounded by pericentriolar material (indicated by CDK5RAP2 or γ-tubulin signals) (Figure 9).^15^^,^^16^

## Limitations

The PVDF membrane used in this protocol exhibits autofluorescence when exposed to blue light, such as 488 nm, which can interfere with fluorescence-based observations. Additionally, the filter membrane may deform during the mounting process, potentially distorting the sample and hindering accurate observations. Moreover, the pore size of the PVDF membrane can limit the capture and staining of certain structures, depending on their size and morphology. Finally, repeated centrifugation steps during immunostaining may physically damage cells or isolated structures.

## Troubleshooting

### Problem 1

The filter membranes become clogged during immunostaining (related to step 1-c and 7-c).

### Potential solution

• Reduce the number of cells.
• Consider the pore sizes of the filter membrane.

### Problem 2

During blocking and antibody incubation, the solution may passively flow-through the filter membrane without centrifugation, causing the sample to dry out (related to step 3-a, 4-a, 5-a, 10-a and 11-a).

### Potential solution

Increase the volume of blocking or antibody solution to avoid the sample drying out during the incubation period.

### Problem 3

Some cells collapse during immunostaining (Figure 7, related to step 13-d).

### Potential solution

• Reduce centrifuge speed and duration.
• Consider fixation conditions. Incubation of cells with fixation solution for hours or pre-fixation of cells prior to centrifugation may help preserve cell morphology.

### Problem 4

Autofluorescence of the filter membrane interferes with the fluorescence observations (related to step 13-d).

### Potential solution

• Avoid using fluorescent antibodies excited with blue light.
• Consider using a confocal microscope to distinguish target signals from autofluorescence.

### Problem 5

Aggregation of antibodies hinders the specific visualization of cells and cellular structures (related to step 13-d).

### Potential solution

• Spin down the antibody solution and use the supernatant.
• Filter the antibodies using centrifugal filter devices (0.1 or 0.22 μm PVDF) and use the flow-through.

### Problem 6

Uneven distribution of cells on the filter membrane (related to step 13-d).

### Potential solution

Use a swing-bucket rotor.

### Problem 7

Specific fluorescent signals for the target proteins are not detected after immunostaining (related to step 13-d).

### Potential solution

• Consider the fixation conditions and the antibodies used.
• Consider antibody concentrations and incubation time.

## Resource availability

### Lead contact

Further information and requests for resources and reagents should be directed to and will be fulfilled by the lead contact, Dr. Shohei Yamamoto (s.yamamoto@mol.f.u-tokyo.ac.jp).

### Technical contact

Technical questions on performing this protocol should be directed to the technical contact, Dr. Shohei Yamamoto (s.yamamoto@mol.f.u-tokyo.ac.jp).

### Materials availability

This study did not generate new unique reagents.

### Data and code availability

This study did not generate any unique datasets or code.

## Acknowledgments

We thank all members of the Kitagawa laboratory for their advice and discussions. This work was supported by JSPS KAKENHI grants from the Ministry of Education, Science, Sports and Culture of Japan (19H05651, 22K20624, 23K14176, 24H02284, and 25K18457); the CREST program (JPMJCR22E1) of the Japan Science and Technology Agency; the Takeda Science Foundation; the Princess Takamatsu Cancer Research Fund; The Uehara Memorial Foundation; the Inamori Foundation; the Astellas Foundation for Research on Metabolic Disorders; The Kishimoto Fund Research Grant from the Senri Life Science Foundation; and The Noguchi Shitagau Research Grant from the Noguchi Institute.

## Author contributions

Conceptualization, M.M., K.K., and S.Y.; methodology, M.M., K.K., and S.Y.; investigation, M.M., K.K., and S.Y.; visualization, M.M., K.K., and S.Y.; writing – original draft, M.M., K.K., and S.Y.; writing – review and editing, M.M., K.K., S.Y., and D.K.; funding acquisition, S.Y. and D.K.; project administration, S.Y. and D.K.; supervision, S.Y. and D.K.

## Declaration of interests

The authors declare no competing interests.
